# Simulation Design of Novel Non-Fluorine Polymers as Electron Transport Layer for Lead-Free Perovskite Solar Cells

**DOI:** 10.3390/polym15224387

**Published:** 2023-11-11

**Authors:** Syed Abdul Moiz, Mohammed Saleh Alshaikh, Ahmed N. M. Alahmadi

**Affiliations:** Device Simulation Laboratory, Department of Electrical Engineering, College of Engineering and Islamic Architecture, Umm Al-Qura University, Makkah 21955, Saudi Arabia; msshaikh@uqu.edu.sa (M.S.A.); anmahmadi@uqu.edu.sa (A.N.M.A.)

**Keywords:** perovskite solar cell, non-fullerene acceptor (NFA), electron transport layer, PEDOT:PSS, Cs_2_AgBi_0.75_Sb_0.25_Br_6_, BT-BIC, BT-LIC, BT-L4F, BT-BO-L4F

## Abstract

Significant progress has been made in the advancement of perovskite solar cells, but their commercialization remains hindered by their lead-based toxicity. Many non-toxic perovskite-based solar cells have demonstrated potential, such as Cs_2_AgBi_0.75_Sb_0.25_Br_6_, but their power conversion efficiency is inadequate. To address this issue, some researchers are focusing on emerging acceptor–donor–acceptor’–donor–acceptor (A-DA’D-A)-type non-fullerene acceptors (NFAs) for Cs_2_AgBi_0.75_Sb_0.25_Br_6_ to find effective electron transport layers for high-performance photovoltaic responses with low voltage drops. In this comparative study, four novel A-DA’D-A-type NFAs, BT-LIC, BT-BIC, BT-L4F, and BT-BO-L4F, were used as electron transport layers (ETLs) for the proposed devices, FTO/PEDOT:PSS/Cs_2_AgBi_0.75_Sb_0.25_Br_6_/ETL/Au. Comprehensive simulations were conducted to optimize the devices. The simulations showed that all optimized devices exhibit photovoltaic responses, with the BT-BIC device having the highest power conversion efficiency (13.2%) and the BT-LIC device having the lowest (6.8%). The BT-BIC as an ETL provides fewer interfacial traps and better band alignment, enabling greater open-circuit voltage for efficient photovoltaic responses.

## 1. Introduction

Hybrid perovskite materials have demonstrated excellent performance over the past several years in the field of solar devices, and as a result their power conversion efficiency increased from a few percent to 27% in a very short period of time [1,2,3]. To further improve the performance of perovskite solar cells, substantial research efforts are being made to address several outstanding problems. Due to these efforts, perovskite solar cells are more advantageous than other types of solar cells in a number of aspects, including cost, weight, flexibility, portability, wide-area application, and low-temperature production [4,5]. Even though perovskite solar cells have made great advances in the laboratory environment, there are still a number of barriers to their general commercialization [6,7].

It is commonly known that the lead-based toxicity, power conversion efficiency, stability, and degradation of perovskite solar cells are the most significant unsolved challenges [8,9]. A highly stable double perovskite class of a Cs_2_AgBiBr_6_ absorber layer has recently been reported as a viable substitute for Pb-based perovskite. As a result, Cs_2_AgBi_0.75_Sb_0.25_Br_6_ is favored for this study due to its various benefits, including being lead-free, non-toxic, extremely stable, and compatible with a variety of transport layers, as reported in the literature [10,11,12].

Despite the perovskite absorber layer, the design architectures, material properties of the charge transport layer, and other design limitations also have serious impacts on the abovementioned problems. Two design architectures, (i) standard n-i-p and (ii) inverted p-i-n, are frequently used for the fabrication of perovskite solar cells, depending on the front electron transport layer or hole transport layer facing the photons for solar cell applications. In both architectures, the perovskite absorber layer is sandwiched between the electron transport layer and the hole transport layer. Additionally, each architecture has unique benefits and drawbacks. Currently, the inverted p-i-n design is used to create the highest-performing photovoltaic devices [13,14,15].

The electron transport layer is one of the most significant functional layers in perovskite solar cells, due to its crucial role in enhancing stability, power conversion efficiency, cost, and consequently overall performance [15,16,17]. There are vital electronic parameters of the electron transport layer that are fundamentally necessary for fine-tuning the effective photovoltaic response, such as the (i) energy bandgap, (ii) electron affinity (LUMO), (iii) ionization energy (HOMO), (iv) molecular packaging, (v) carrier mobilities, (vi) reorganization energy, etc. The great majority of polymer and perovskite solar cells used for photovoltaic applications include an electron transport layer composed of fullerene materials as PC_60_BM ([6,6]-phenyl C61-butyric acid methyl ester) and/or PC_70_BM ([6,6]-phenyl-C71-butyric acid methyl ester) [18,19,20]. The fullerene-based electron transport layer, unfortunately, has several drawbacks, such as (i) poor optical absorption, especially in the near-infrared and visible range; (ii) thermal instability; (iii) photochemical instability; (iv) restricted tuneability, etc. [21,22].

**Figure 1:**
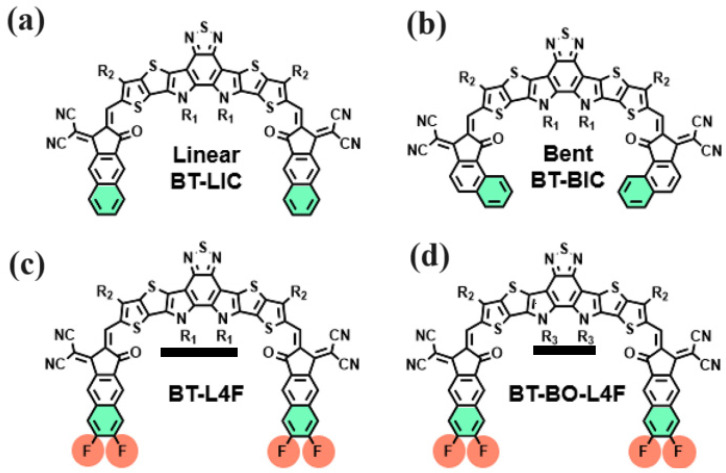
The molecular structure of (**a**) BT-LIC, (**b**) BT-BIC, (**c**) BT-L4F, and (**d**) BT-BO-L4F is used as a non-fluorine electron transport layer.

Designing a non-fullerene-based (NFA) electron transport layer might potentially overcome the drawbacks of the fullerene-based electron transport layer. The stability, tuneability, and optical absorption can all be improved by easily adjusting the chemical molecular structure, highest occupied molecular orbital (HOMO), and lowest unoccupied molecular orbital (LUMO) of a non-fullerene-based electron transport layer over a reasonably wide range [23,24,25]. The design of NFAs that are reported often falls into one of two categories. One choice is the acceptor–donor–acceptor (A-D-A)-type molecule, which has a simple manufacturing process and an energy level that is easily adjusted. The other, the A-DA’D-A-type molecule, on the other hand, is supported by a greater short-circuit current density (J_SC_) and wider absorption, thanks to its bigger conjugated plane and enhanced intramolecular charge transfer (ICT). It is quite interesting to design organic/polymer electron transport materials, notably with the help of A-DA’D-A-type NFAs and their variants [26,27,28,29,30,31,32,33,34,35]. All these design parameters can be greatly tuned for an effective electron transport layer in a perovskite solar cell by making use of various physiochemical methodologies [36,37,38,39]. On the basis of these physiochemical methodologies, four novel A-DA’D-A types of NFAs as electron transport layers have recently been reported, which are the end-group derivatives of Y5 and Y6 materials and named linear as BT-LIC and bent as BT-BIC, BT-L4F, and BT-BO-L4F [40,41,42,43], as shown in Figure 1. All of these, (i) BT-LIC, (ii) BT-BIC, (iii) BT-L4F, and (iv) BT-BO-L4F, are highly novel materials for the electron transport layer, with very little and nearly negligible information available in the reported literature.

In most cases, it may be difficult to dope polymers for electron/hole transport layers (ETLs) at higher concentrations. This problem is caused by many variables, including (i) solubility and compatibility, (ii) aggregation and phase separation, (iii) doping-induced defects, and (iv) doping process restrictions. It is crucial to note that although high doping concentrations in polymer transport layers might be difficult, they are not always required. The maximum doping of the electron/hole transport layer is still reported in the literature at a value of 10^20^ cm^−3^. For this reason, in our simulation of the suggested solar cell, we employ a maximum doping density of up to 10^20^ cm^−3^ for both electron and hole transport layers [44,45].

**Figure 2:**
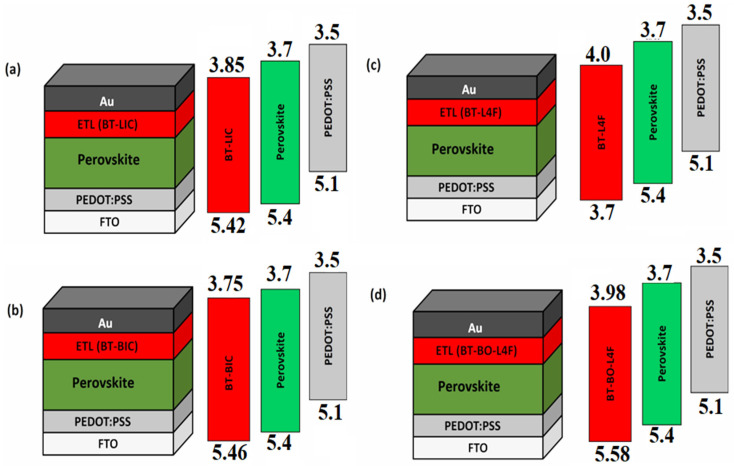
Design architecture of the proposed perovskite solar cells with their energy band diagrams: (**a**) FTO/PEDOT:PSS/Cs_2_AgBi_0.75_Sb_0.25_Br_6_/BT-LIC/Au (device BT-LIC), (**b**) FTO/PEDOT:PSS/Cs_2_AgBi_0.75_Sb_0.25_Br_6_/BT-BIC/Au (device BT-BIC), (**c**) FTO/PEDOT:PSS/Cs_2_AgBi_0.75_Sb_0.25_Br_6_/BT-L4F/Au (device BT-L4F), and (**d**) FTO/PEDOT:PSS/Cs_2_AgBi_0.75_Sb_0.25_Br_6_/BT-BO-L4F/Au (device BT-BO-L4F).

In this study, the p-i-n-type perovskite solar cells were chosen, as they demonstrate relatively higher efficiency and ease of fabrication, as discussed above [46,47,48,49,50]. Similarly, PEDOT: PSS (poly(3,4-ethylenedioxythiophene) polystyrene sulfonate)) as the hole transport layer and Cs_2_AgBi0_.75_Sb_0.25_Br_6_ as the perovskite layer were selected due to many advantages, such as being lead-free, highly stable, compatible with both transport layers, etc. [51,52]. Figure 2 shows the design architecture of the four proposed devices with their energy band diagrams, namely (i) FTO/PEDOT: PSS/Cs_2_AgBi_0.75_Sb_0.25_Br_6_/BT-LIC/Au (device BT-LIC), (ii) FTO/PEDOT: PSS/Cs_2_AgBi_0.75_Sb_0.25_Br_6_/BT-BIC/Au (device BT-BIC), (iii) FTO/PEDOT: PSS/Cs_2_AgBi_0.75_Sb_0.25_Br_6_/BT-L4F/Au (device BT-L4F), and (iv) FTO/PEDOT: PSS/Cs_2_AgBi_0.75_Sb_0.25_Br_6_/BT-BO-L4F/Au (device BT-BO-L4F). The main goal of this study is to comprehensively investigate, optimize, and compare the devices as a function of the above hole transport layer to determine the different design parameters that can offer the maximum power conversion efficiency. In order to maintain simplicity, this study focuses exclusively on the analysis and comparison of the thickness and doping density of the electron transport layer (ETL). The manuscript grows excessively lengthy in other cases. However, future study endeavors may involve the utilization of optimal thickness and doping values, as well as a comprehensive analysis and explanation of the various properties related to the electron transport layer.

## 2. Device Models for Simulation

The software for modeling solar cells typically solves a set of coupled differential equations for semiconductor devices using conventional mathematical techniques. The general photovoltaic responses of solar cells, such as short-circuit current, open-circuit voltage, fill factor, and power conversion efficiency, are identified using the solutions of these equations [53,54,55,56,57]. Detailed information about these models can be found in our previously published results [7].

The SCAPS 1D (version 3.3.10) software employs a drift-diffusion model for the purpose of computing the energy levels within the band diagram of a photovoltaic cell. These equations incorporate various parameters, including carrier production, recombination, and transport processes. The SCAPS 1D software is capable of solving Poisson’s equation, which establishes a relationship between the electric field and the charge distribution present in a solar cell. The aforementioned equation incorporates the impact of the electrostatic potential on the energy levels depicted in the band diagram. SCAPS 1D utilizes the derived electric field and charge distribution to ascertain the band offsets and energy levels at different locations within the band diagram of the solar cell.

## 3. Simulation Software

A solar cell’s photovoltaic response may be estimated using the mathematical equations mentioned above, which are the foundation of any photovoltaic modeling software. In theory, simulation software is a tool that allows users to foretell the output response of solar devices without performing actual testing in a variety of settings. Simulation software can reasonably predict the experimental results of a solar cell [57,58,59,60]. The conditions must be met by modeling software to accurately forecast the output of photovoltaic responses accurately. Numerous researchers assert that SCAPS 1D is extremely trustworthy software that satisfies many of the criteria listed in the references [61,62,63,64] and may be used to simulate a wide range of photovoltaic processes.

## 4. Simulation Steps

In general, the SCAPS 1D simulation steps are a list of feasible actions that must be executed. The following is a list of the simulation processes that are necessary to apply to the suggested devices in order to obtain the highest power conversion efficiency.

Step 1 Start of simulation: Define the environment, geometry, and physical parameters of all the device’s layers according to Table 1.
Step 2 Extraction of simulation parameters: Extract the input physical and material characteristics for the perovskite absorber layer, the hole transport layer (HTL), and the electron transport layer (ETL) using the literature as a guide and updated in Table 1 [63,64,65,66,67,68,69,70,71].
Step 3 Estimation of ranges for different parameters: Propose the range of thickness and doping density for each layer of (i) device BT-LIC, (ii) device BT-BIC, (iii) device BT-L4F, and (iv) device BT-BO-L4F from the literature.
Step 4 Thickness optimization of HTL: Determine the optimal thickness of PEDOT: PSS for each device as a hole transport layer through a series of simulations, which gives the maximum power conversion efficiency and quantum efficiency (QE). After that, update with the optimal thickness of PEDOT: PSS for further simulations.
Step 5 Determination of PV parameters as a function of HTL thickness: Determine the photovoltaic parameters such as open-circuit voltage, short-circuit current, fill factor, and power conversion efficiency of each device as a function of PEDOT: PSS thickness.
Step 6 Thickness optimization of perovskite absorber layer: Determine the optimal thickness of the perovskite absorber layer (Cs_2_AgBi_0.75_Sb_0.25_Br_6_) for each device as an absorber layer through a series of simulations, which gives the maximum power conversion efficiency and quantum efficiency. Then, update with the optimal thickness of the absorber for further simulations.
Step 7 Determination of PV parameters as a function of perovskite thickness: Determine the photovoltaic parameters such as open-circuit voltage, short-circuit current, fill factor, and power conversion efficiency of each device as a function of absorber thickness. But for simplicity, only the photovoltaic parameters of a highly efficient device are shown and discussed.
Step 8 Thickness optimization of ETL: Determine the optimal thickness of the ETL for each device ((i) BT-LIC, (ii) BT-BIC, (iii) BT-L4F, and (iv) BT-BO-L4F) through a series of simulations, which gives the maximum power conversion efficiency and quantum efficiency. After that, update with the optimal thickness of the ETL for further simulations.
Step 9 Determination of PV parameters as a function of optimized ETL thickness: Determine the photovoltaic parameters such as open-circuit voltage, short-circuit current, fill factor, and power conversion efficiency of each device as a function of electron transfer layer thickness.
Step 10 Determination of PV parameters as a function of ETL doping: Determine the photovoltaic parameters such as open-circuit voltage, short-circuit current, fill factor, and power conversion efficiency of each device as a function of ETL doping density.
Step 11 Determination of PV response and parameters of the optimized devices: Determine the photovoltaic current–voltage response and other photovoltaic parameters of all the optimized devices such as open-circuit voltage, short-circuit current, fill factor, and power conversion efficiency of each device as a function of ETL doping density.
Step 12 Determination of QE response of the optimized devices: Determine the quantum efficiency of all the optimized devices.
Step 13 End of simulation.

**Table 1:** Random simulation parameters such as thickness and doping are used for the novel non-fluorine polymer acceptor transport layer, while other simulation parameters for given materials are taken from the given references.

Photovoltaic Parameters	PEDOT:PSS	Perovskite Cs_2_AgBi_0.75_Sb_0.25_Br_6_	BT-LIC	BT-BIC	BT-L4F	BT-BO-L4F
Thickness (nm)	50	500	100	100	100	100
Energy Bandgap (E_g_, eV)	2.2	1.8	1.57	1.73	1.58	1.6
Electron Affinity (X, eV)	2.9	3.58	3.85	3.73	4	3.98
Dielectric Permittivity ($\epsilon_{r}$)	3.0	6.5	3.5	3.5	3.5	3.5
Effective Density of States at Conduction Band (N_c_, cm^−3^)	2.2 × 10^15^	2.2 × 10^18^	1 × 10^20^	1 × 10^20^	1 × 10^20^	1 × 10^20^
Effective Density of States at Valence Band (N_v_, cm^−3^)	1.8 × 10^18^	1.8 × 10^19^	1 × 10^20^	1 × 10^20^	1 × 10^20^	1 × 10^20^
Hole Thermal Velocity (V_h_, cm/s)	1 × 10^7^	1 × 10^7^	1 × 10^7^	1 × 10^7^	1 × 10^7^	1 × 10^7^
Electron Thermal Velocity (V_e_, cm/s)	1 × 10^7^	1 × 10^7^	1 × 10^7^	1 × 10^7^	1 × 10^7^	1 × 10^7^
Electron Mobility ($\mu_{e}$, cm^−2^/V.s)	10	2	1 × 10^−4^	1 × 10^−4^	1 × 10^−4^	1 × 10^−4^
Hole Mobility ($\mu_{h}$, cm^−2^/V.s)	10	2	1 × 10^−4^	1 × 10^−4^	1 × 10^−4^	1 × 10^−4^
Uniform Shallow Donor Doping (N_d_,,cm^−3^)	-	-	1 × 10^16^	1 × 10^16^	1 × 10^16^	1 × 10^16^
Uniform Shallow Acceptor Doping (N_a_, cm^−3^)	10^15^	-	1 × 10^16^	-	-	-
Defect Density (N_t_, cm^−3^)	10^14^	10^14^	10^14^	10^14^	10^14^	10^14^
Reference	[65,66,67,68,69]	[70,71]			[72,73,74]	

## 5. Simulation Material Parameters

For the optimization of each layer of a perovskite solar cell, the proper selection of material parameters for each layer is essential. As the electron transport materials (i) BT-LIC, (ii) BT-BIC, (iii) BT-L4F, and (iv) BT-BO-L4F are novel, an extensive amount of related literature was reviewed to extract the simulation parameters, while for PEDOT:PSS and Cs_2_AgBi_0.75_Sb_0.25_Br_6_ materials, the parameters were selected from highly reliable references, as listed in Table 1.

Both the proposed perovskite absorber and the polymer-based transport layer offer an inherently high density of defects. In most cases, these electronic defects are grown during the crystal growth or fabrication process due to uncontrolled impurities, inconsistencies in polymerization or crystal growth, thin-film deposition processes, and other environmental parameters [74,75,76,77]. Therefore, a defect density of 10^14^ cm^2^ is introduced into the bulk region of the absorber, hole transport layer, and electron transport layer, as indicated in Table 1.

## 6. Results and Discussion

### 6.1. Thickness Optimization of the Hole Transport Layer

Generally, for any type of solar cell, the thickness of the hole transport layer (HTL) plays a crucial role in improving the overall performance of the solar cell. The effectiveness of the charge transfer process between the perovskite absorber layer and the electrode can be considerably impacted by the thickness of the hole transport layer. To obtain the highest possible efficiency in solar cells, it is essential to optimize the thickness of the hole transport layer.

**Figure 3:**
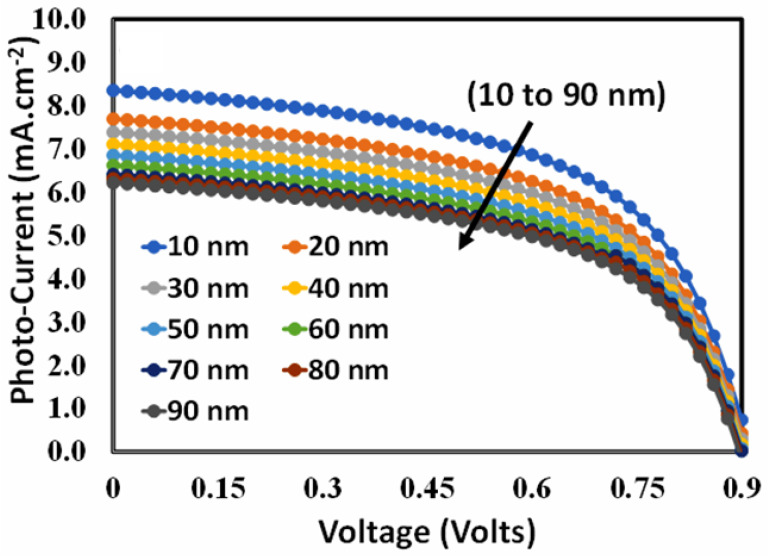
The photo current–voltage responses of the proposed perovskite solar cell for devices where absorber and ETL (device D) thickness are randomly selected (optimization will be performed in a later stage) but the thickness of PEDOT:PSS is varied from 10 nm to 90 nm.

Therefore, determining the optimized thickness of the hole transport layer is a design challenge for perovskite solar cells. In order to determine the optimal thickness of the hole transport layer, each of the four devices was simulated as a function of hole transport layer thickness, and a very similar photovoltaic response was observed. For simplicity, randomly elected device D (BT-LIC) responses are shown in Figure 3. The photo current–voltage response may be used to estimate the thickness of the hole transport layer for perovskite solar cells with the maximum feasible efficiency. Therefore, the photovoltaic responses of device D are depicted in Figure 3 as functions of the thickness of the hole transport layer, which ranges from 10 nm to 90 nm. Based on Figure 3, it can be seen that device D exhibits respectable photovoltaic behavior. However, when the thickness increases, the photovoltaic responsiveness suffers, as the parameters are severely degraded.

**Figure 4:**
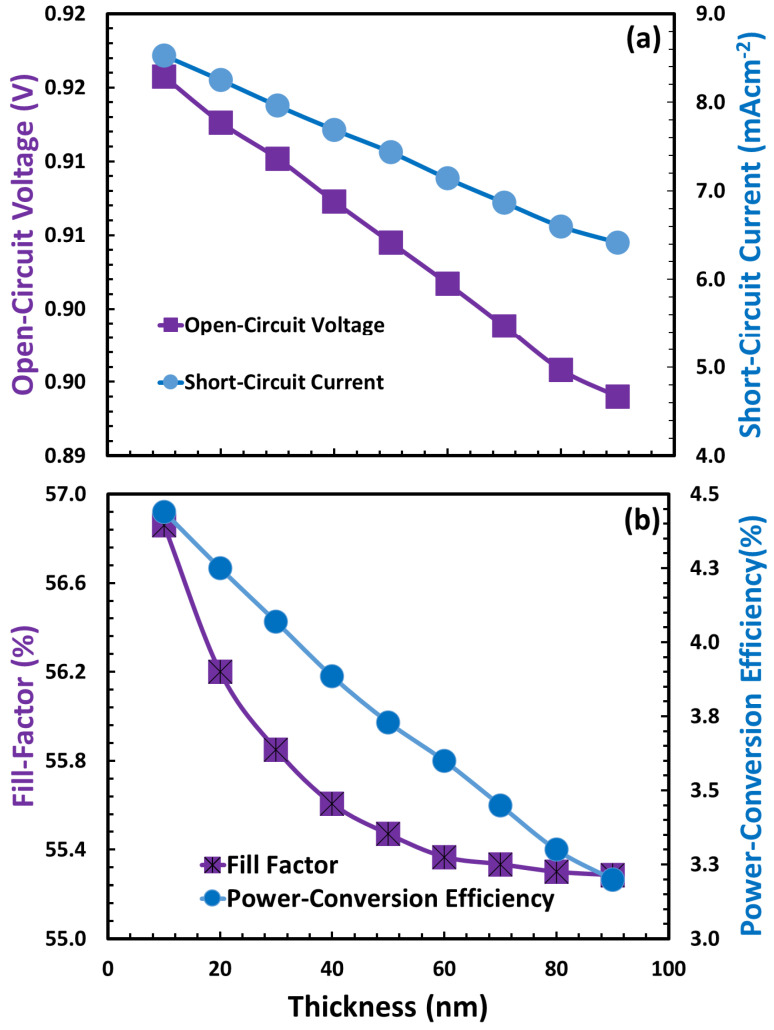
The photovoltaic parameters (**a**) open-circuit voltage (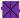), short-circuit current (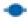), (**b**) fill factor (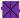), and power conversion efficiency (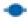) of device D as a function of the thickness of the PEDOT:PSS as hole transport layer.

The photovoltaic parameters were calculated, and the results are shown in Figure 4 as a function of PEDOT: PSS thickness. Therefore, the photovoltaic parameters, such as (i) open-circuit voltage, short-circuit current (Figure 4a), (ii) fill factor, and (iv) power conversion efficiency (Figure 4b), follow more or less very similar trends, where all these parameters are severely degraded. According to a previous report, PEDOT: PSS, like many other organic/polymer semiconductors, inherently has a large number of traps that serve as recombination centers. The density of traps exponentially rises as PEDOT:PSS thickness increases, and photovoltaic characteristics rapidly deteriorate [78,79,80]. As power conversion efficiency is a decisive parameter and it is maximum at 10 nm for each device (here only device D is shown), it can be justified that 10 nm is the optimum thickness of the hole transport layer for each device. This is because the optimal PEDOT:PSS doping density for each proposed device is obtained at 10^20^ cm^−3^ through simulation, which is quite comparable to our previous results [7,61,62,68,69,70].

**Figure 5:**
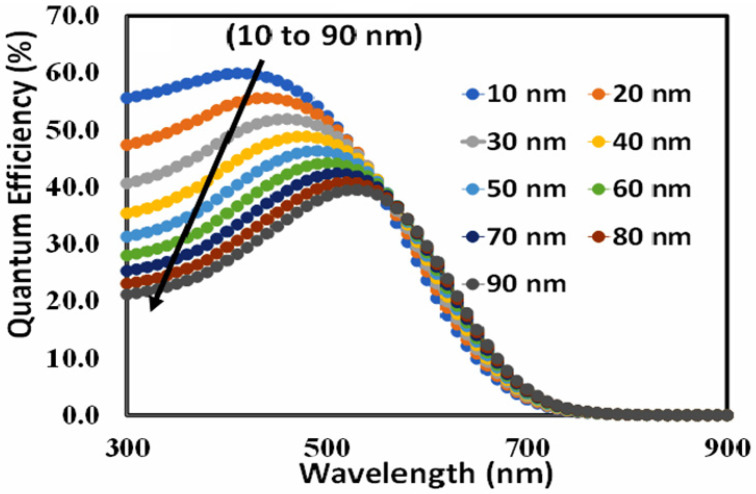
The external quantum efficiency responses of the proposed perovskite solar device D (BT-BO-L4F) as a function of incident photon wavelengths from 300 to 900 nm as a function of PEDOT:PSS thickness from 10 to 90 nm.

For a perovskite-type solar cell, the optimal thickness of the hole transport layer can also be estimated using external quantum efficiency (EQE). The external quantum efficiency depends on the wavelength of the incident light and is often stated in terms of percentage. Figure 5 shows the external quantum efficiency of device D (BT-BO-L4F) as a function of the thickness of PEDOT: PSS. It also confirms that as thickness increases, the area under the QE curve also decreases. It clearly demonstrates that the total charges collected at the respective electrodes also decrease, and hence it can be inferred that more and more recombination will take place at the higher thickness of PEDOT:PSS. Both power conversion efficiency and external quantum efficiency are decisive factors in the selection of the thickness of PEDOT:PSS for efficient photovoltaic response, and maximum power conversion efficiency and quantum efficiency is observed at 10 nm of PEDOT:PSS, so it can be justified that 10 nm is the most optimum thickness of PEDOT:PSS for the proposed devices.

### 6.2. Thickness Optimization of the Absorber Layer

To increase the photo conversion efficiency of perovskite solar cells, the thickness of the absorber layer of Cs_2_AgBi_0.75_Sb_0.25_Br_6_ must be optimized. The ideal thickness of the perovskite absorber layer depends on a number of parameters, including the composition of the perovskite material, the design of the photovoltaic device, and the thin-film deposition process. Thick perovskite absorber layers can boost photon absorption, and on the other hand, thin perovskite absorber layers can decrease recombination losses and lead to improved power conversion efficiency [81,82]. In the literature, both experimental methods and device simulation modeling can be used to determine the optimal thickness of the perovskite absorber layer.

As discussed above, the optimization of perovskite layer thickness is a critical parameter in determining the performance of a solar cell. In order to achieve a suitable balance between light absorption and charge carrier extraction, it is often necessary to optimize the thickness of the active layer. Increased thickness of the active layer facilitates enhanced light absorption due to the greater availability of material for photon interaction. However, a thick active layer increases the carrier recombination. In thicker active layers, the distance that charge carriers need to travel to reach the electrodes increases. Conversely, when the active layer is very thin, it may lead to inadequate light absorption, thus leading to less production of photocurrent. Similarly, a thin active layer may also limit the ability to extract charge carriers efficiently, leading to poor charge collection and reduced overall performance.

**Figure 6:**
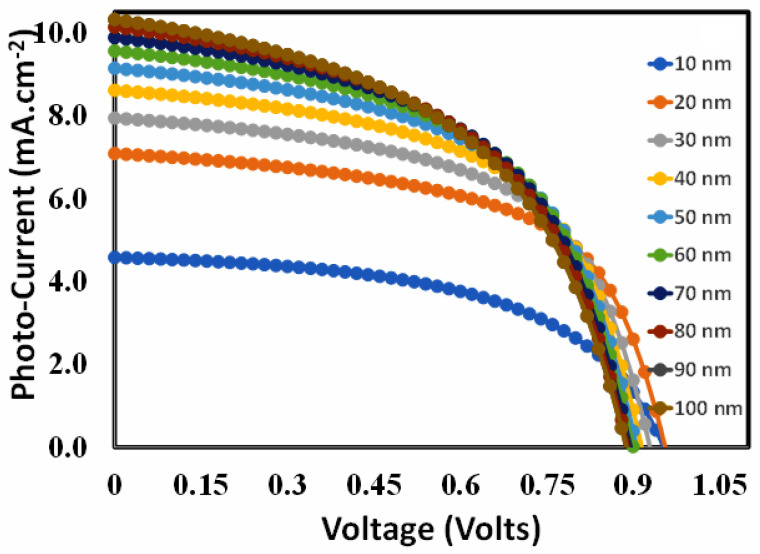
The photo current–voltage responses of the proposed perovskite solar cell for device D while the thickness of the absorber layer (Cs_2_AgBi_0.75_Sb_0.25_Br_6_) varied from 10 nm to 100 nm.

**Figure 7:**
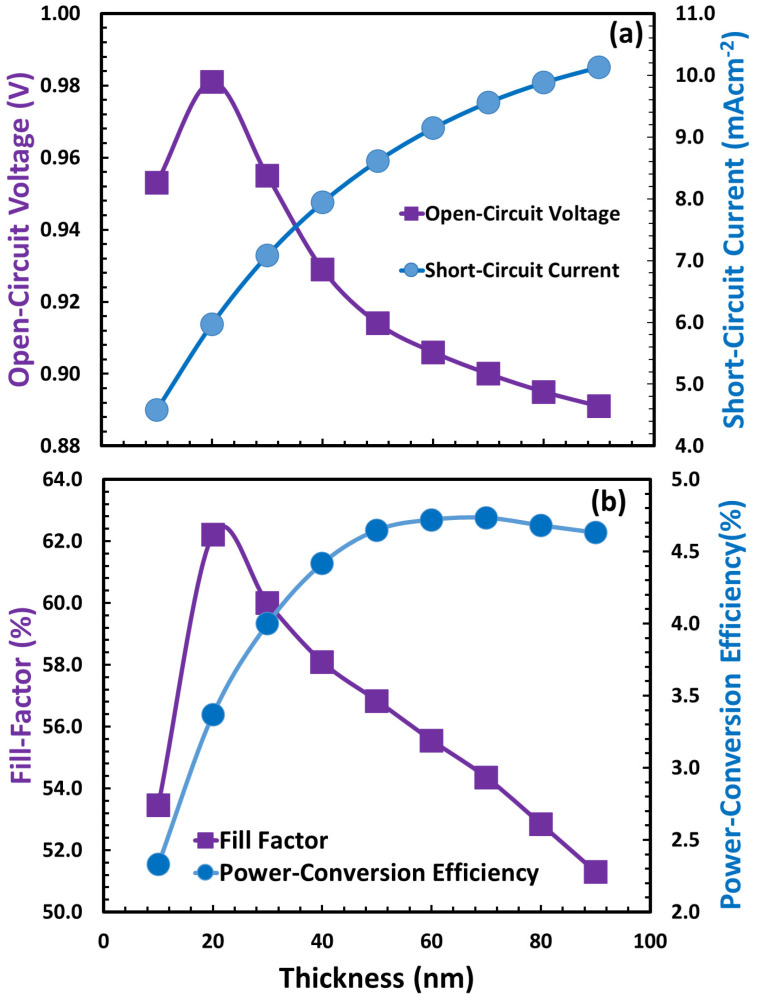
The responses of photovoltaic parameters (**a**) open-circuit voltage and short-circuit current and (**b**) fill factor and power conversion efficiency of the proposed perovskite solar cell for device D while the thickness of the absorber layer (Cs_2_AgBi_0.75_Sb_0.25_Br_6_) is varied from 10 nm to 100 nm.

Here, the optimal thickness of the perovskite absorber Cs_2_AgBi_0.75_Sb_0.25_Br_6_ layer was estimated using simulation techniques. In order to start the simulation, the absorber layer was initialized with random values extracted from Table 1. The photovoltaic response of device D is depicted in Figure 6 as a function of absorber layer thickness from 10 nm to 100 nm. According to the device’s photovoltaic response, unlike the thickness of the hole transport layer, the photovoltaic response is degraded at lower absorber layer thicknesses, increases up to when the absorber layer reaches its maximum photovoltaic response, and then again gradually decreases; the above-discussed responses are more clearly observed in Figure 7.

Figure 7a,b illustrate the photovoltaic parameters of each device as a function of absorber layer thickness, including open-circuit voltage, short-circuit current, fill factor, and power conversion efficiency. Complex photovoltaic parameter patterns are observed, where the open-circuit voltage and fill factor climb suddenly, peak, and then rapidly deteriorate. While the short-circuit current and power conversion efficiency behavior are different for a device as a function of absorber layer thickness, both of these photovoltaic parameters increase gradually, and the power conversion efficiency in particular reaches its maximum at 70 nm thickness of the absorber layer before beginning to slowly decline.

**Figure 8:**
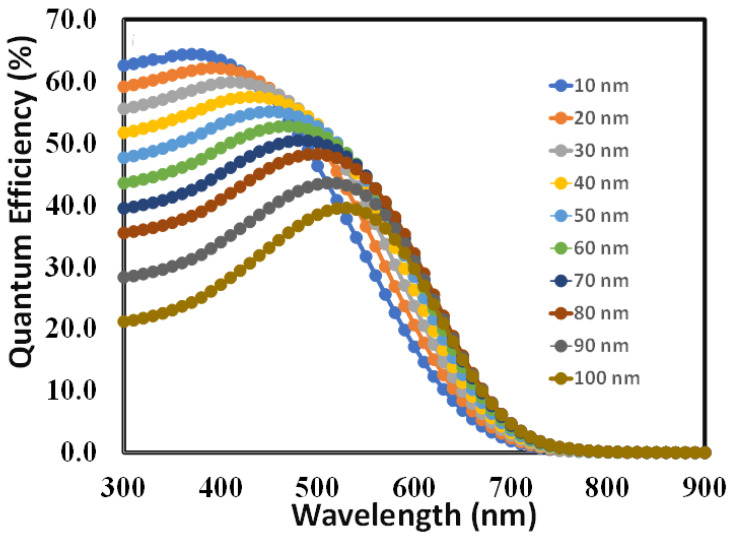
The quantum efficiency responses of the proposed perovskite solar cell for device D, where the thickness of the absorber layer (Cs_2_AgBi_0.75_Sb_0.25_Br_6_) is varied from 10 nm to 100 nm.

Figure 8 illustrates the quantum efficiency response of the proposed perovskite solar cell for devices where the thickness of the absorber layer (Cs_2_AgBi_0.75_Sb_0.25_Br_6_) is varied from 10 nm to 100 nm and the thickness of the PEDOT:PSS is optimized at 10 nm. It is not clearly observed from the figure which thickness of the absorber layer gives the maximum area under the quantum efficiency curve. However, with the help of software calculations, it is found that at the thickness of 70 nm of the absorber layer, the device gives the maximum quantum efficiency, which further supports the finding that 70 nm is the optimum thickness of the absorber layer.

In a Cs_2_AgBi_0.75_Sb_0.25_Br_6_-based solar cell, the redshift of the band edge impacts its quantum efficiency, which is influenced by the thickness of the Cs_2_AgBi_0.75_Sb_0.25_Br_6_ absorber layer, as shown Figure 8. Increasing the absorber layer thickness causes the band boundary to shift towards longer wavelengths, resulting from changes in the electrical structure and optical properties of the perovskite absorber layer. The relationship between electronic structure, optical properties, and thickness can be summarized as follows:

1. Thicker absorber layers enhance absorption efficacy by capturing more photons, increasing the likelihood of photon absorption and charge carrier generation.
2. The thickness of the absorber layer affects charge carrier extraction. Excessive thickness can lead to increased recombination or trapping of charge carriers, reducing the quantum efficiency.
3. Thicker absorber layers may experience more light scattering or reflection at interfaces, resulting in a loss of absorbed photons and potentially impacting the quantum efficiency.
4. Thin absorber layers may exhibit limited charge transport and higher recombination rates, while excessively thick layers can impede charge extraction due to longer carrier transit times, both affecting the overall efficiency.

Therefore, to optimize the perovskite absorber, it is crucial to find the right thickness of the Cs_2_AgBi_0.75_Sb_0.25_Br_6_ absorber layer that balances effective charge carrier extraction with efficient photon absorption. Thus, it can be assumed that 70 nm is the optimal thickness of the perovskite absorber layer that provides a balance between the above-stated variables.

### 6.3. Thickness Optimization of Electron Transport Layer

Another critical design factor for producing highly efficient perovskite solar cells is the thickness optimization of the electron transport layer. Thickness optimization, particularly for polymer-based electron layers, is very important because it affects several physical, material, and photovoltaic parameters.

(a) Device architecture: The optimal electron transport layer thickness may vary according to the specific device architecture, such as whether the solar cell has a planar or mesoporous structure.
(b) Material characteristics: The ideal thickness for effective charge transfer and extraction can be influenced by the electron transport layer’s material characteristics, such as (i) electron transport, (ii) series resistance, (iii) shunt resistance, (iv) carrier lifetime, (v) electron mobility, (vi) electron density, (vii) recombination losses, (viii) charge extraction efficiency, (ix) optical absorption, and (x) interface energy level alignment, among others.
(c) Thin-film deposition method: There are several thin-film deposition methods that may be used to manage the electron transport layer’s thickness, and each has advantages and limitations of its own. Spin coating, for instance, is a very cheap and simple method.
(d) Post-deposition method: By modifying the thickness of the electron transport layer using post-deposition techniques like solvent or annealing, the morphology and interface properties of the electron transport layer may be altered.
(e) Photovoltaic performance matrices: Depending on the device’s individual performance metrics, such as power conversion efficiency, short-circuit current, and fill factor, the ideal electron transport layer thickness may vary. These metrics may all be influenced by the electron transport layer’s thickness.
(f) Trade-offs: When determining the ideal electron transport layer thickness, it is frequently necessary to compromise between several device characteristics, such as reducing leakage currents while increasing charge transfer efficiency.
(g) Interaction with other layers: The electron transport layer’s thickness can have an impact on how the electrode, absorber layer, and hole transport layer interact with other layers in the device.
(h) Energy level alignment: The energy level alignment between the ETL and the perovskite layer exhibits some differences. Such differences can lead to a modification of the energy levels inside the valence band (VB) and conduction band (CB) at the interface separating the electron transport layer (ETL) and the perovskite material. The determination of the energy level offset plays a crucial role in driving the process of charge transfer and has a significant impact on the efficiency of charge extraction and hence the efficiency of solar cells. This might make the optimization procedure more challenging.

Overall, the complicated interactions between these variables make optimizing the electron transport layer thickness a difficult iterative process that frequently calls for simulation methods.

**Figure 9:**
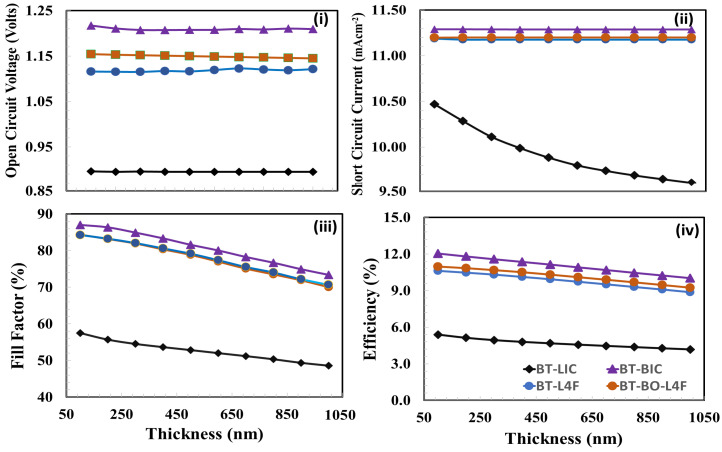
The photovoltaic parameters (**i**) open-circuit voltage, (**ii**) short-circuit current, (**iii**) fill factor, and (**iv**) power conversion efficiency of all devices, (a) BT-LIC, (b) BT-BIC, (c) BT-L4F, and (d) BT-BO-L4F, as a function of the thickness of the electron transport layer.

The photovoltaic parameters (i) open-circuit voltage, (ii) short-circuit current, (iii) fill factor, and (iv) power conversion efficiency are shown in Figure 9 as functions of the thickness of the electron transport layer for the devices (a) A (BT-LIC), (b) B (BT-BIC), (c) C (BT-L4F), and (d) D (BT-BO-L4F). Figure 9 demonstrates that although the open-circuit voltage varies for each device, it does not change relatively as the thickness of the electron transport layer increases. This is because the open-circuit voltage is generally determined by the energy bandgap offset, and its value is generally unaffected by the thickness of the electron transport layer [83,84], as observed in Figure 9. In addition, Figure 9 shows the relative short-circuit responses for the devices as functions of the thickness of the electron transport layer. The figure demonstrates that all devices behave in a consistent manner, with the short-circuit current reducing as the thickness of the electron layer grows. Device A’s behavior stands out, whereas other devices’ rates of decrease are very insignificant. Similarly, when the thickness of the electron transport layer increases, the fill-factor behavior of all devices likewise deteriorates. This could be because the devices experience increasing series resistance and recombination losses as the thickness of the electron layer rises. The power conversion efficiency responses, which are the resultant behavior of open-circuit voltage, short-circuit current, and fill-factor responses, also behave in a very similar way, while the power conversion efficiency deteriorates as a function of electron transport layer thickness. The figure clearly demonstrates that device BT-LIC is sharply degraded, while device BT-BIC shows a relatively excellent power conversion efficiency response as a function of electron transport layer thickness. Power conversion efficiency is the most important parameter, and all devices show maximum power conversion efficiency at 25 nm. Thus, it can be inferred that 25 nm is the most optimal thickness of the corresponding electron transport layer for each device.

### 6.4. Doping Density Optimization of the Electron Transport Layer

The electron transport layer’s doping density can have a big influence on the electrical conductivity of solar cells, charge carrier mobility, and overall power conversion efficiency. Usually, organic or polymeric compounds with strong electron-withdrawing groups and significant electron affinities are used as dopants to dope polymer-based electron transport layers [85,86]. High doping of the electron transport layer (ETL) can improve the power conversion efficiency of a perovskite solar cell by lowering the series resistance and electrode-transport layer interface losses, but on the other hand, excessive doping can increase charge carrier recombination and degrade the device’s overall efficiency. Therefore, doping optimization of the electron transport layer is crucial for the efficient design of the proposed perovskite solar cells.

**Figure 10:**
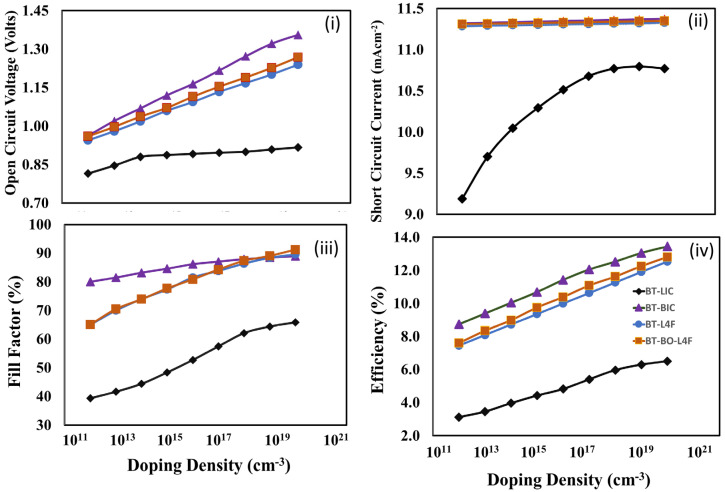
The photovoltaic parameters (**i**) open-circuit voltage, (**ii**) short-circuit current, (**iii**) fill factor, and (**iv**) power conversion efficiency of the proposed devices, (a) device BT-LIC, (b) device BT-BIC, (c) device BT-L4F, and (d) device BT-BO-L4F, as a function of the doping density of the electron transport layer.

Therefore, the photovoltaic parameters (a) open-circuit voltage, (ii) short-circuit current, (iii) fill factor, and (d) power conversion efficiency were estimated through simulation, and the results are shown in Figure 10 as functions of the doping density of the electron transport layer for the devices (a) A (BT-LIC), (b) B (BT-BIC), (c) C (BT-L4F), and (d) D (BT-BO-L4F). The figure shows that all the photovoltaic parameters, open-circuit voltage, short-circuit current, fill factor, and power conversion efficiency, are improving as the doping density is increased in the corresponding electron transport layer. The thickness of the electron transport layer is already optimized, and it can be assumed that the electron transport layer offers very little recombination losses; therefore, the reduction in resistivity as a function of increasing doping density is the dominating factor for each device. As power conversion efficiency is a decisive parameter and it is maximum at 10^20^ cm^−3^ for each device, it can be justified that 10^20^ cm^−3^ is the optimum doping of the electron transport layer for each device.

### 6.5. Photo Current–Voltage Response of the Optimized Devices

**Figure 11:**
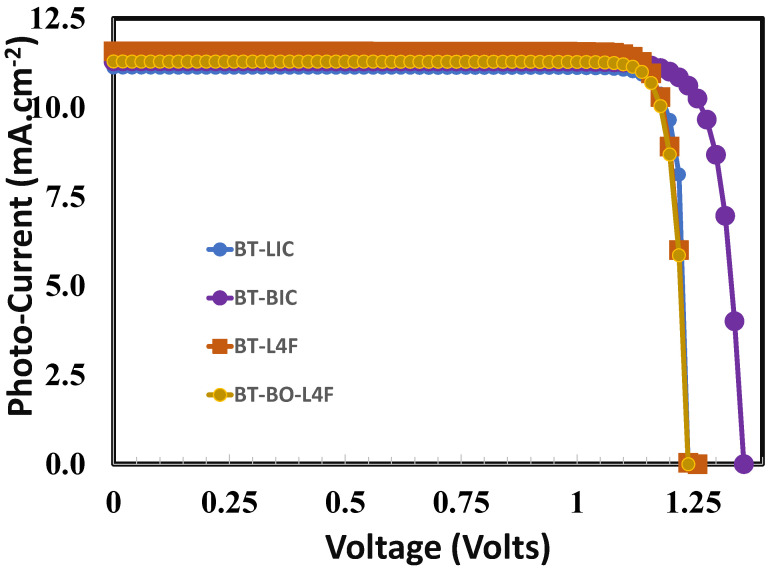
The photo current–voltage responses of the proposed perovskite solar cells for the device (i) BT-LIC, (ii) BT-BIC, (iii) BT-L4F, and (iv) BT-BO-L4F respectively.

The influence of the electron transport layer on the overall performance of perovskite solar cells may be further investigated by using the photo current–voltage responses of the optimized perovskite solar cells. In Figure 11, the typical photo current–voltage responses of the perovskite solar cells are observed. The figure offers important information about its photovoltaic performance parameters such as open-circuit voltage, short-circuit current, fill factor, and power conversion efficiency. Table 2 displays all these photovoltaic parameters as estimated from Figure 11 for each device. The figure and Table 2 clearly reveal that all devices perform differently, while device B (BT-BIC) has the best photovoltaic response (power conversion efficiency ~13.2%), and device A (BT-LIC) has the worst photovoltaic response (power conversion efficiency ~6.8%). It is also observed that the improvements in open-circuit voltage as well as fill factor are relatively higher for device B. This clearly demonstrates that the photovoltaic device B fabricated with BT-BIC as an electron transport layer may manage the optimum build-in potential and excellent interface quality, which in turn improve the overall charge collection and charge transport and suppress recombination losses [87,88,89] compared to the other electron transport layers for devices A, C, and D.

### 6.6. External Quantum Efficiency Response of the Optimized Devices

**Figure 12:**
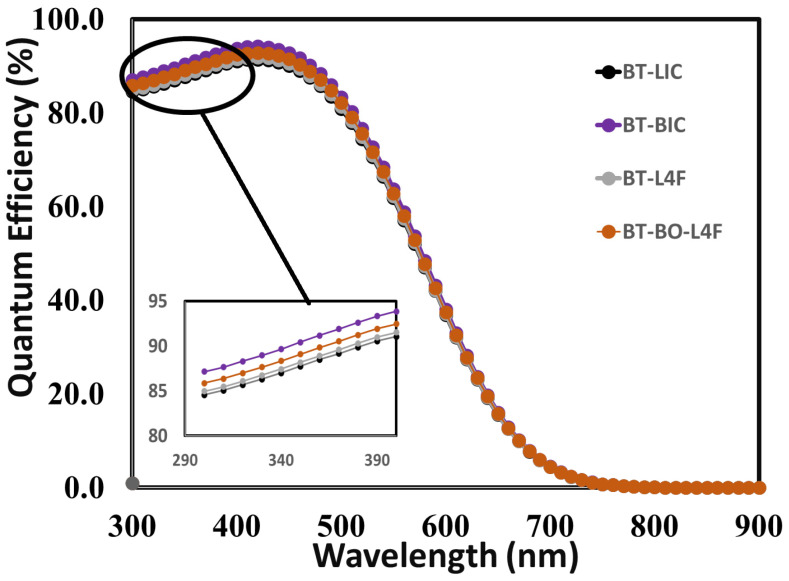
The external quantum efficiency responses of the proposed perovskite solar cells for the device (i) BT-LIC, (ii) BT-BIC, (iii) BT-L4F, and (iv) BT-BO-L4F as a function of incident photon wavelengths from 300 to 900 nm. The inset of the figure shows the magnified responses of all the devices for the incident photon wavelength range from 300 to 390 nm.

The external quantum efficiency responses of the fully optimized devices may be used to further examine the impact of the electron transport layer on the overall performance of perovskite solar cells. The external quantum efficiency can be defined as the fraction of the total number of collected charge carriers at the electrodes to the total number of incident photons [90]. Mathematically, external quantum efficiency (QE) can be defined as a function of either energy E or wavelength (*λ*), and therefore the relation between quantum efficiency and short-circuit current (*Jsc*) can be expressed as

(1) $$J_{SC}=\;q\;\int \emptyset \left(\lambda \right)\;QE\left(\lambda \right)d\lambda$$

where φ(*λ*) is the incident flux of photons per unit wavelength. Figure 12 shows the external quantum efficiency responses of (i) device BT-LIC, (ii) device BT-BIC, (iii) device BT-L4F, and (iv) device BT-BO-L4F as a function of incident photon wavelengths from 300 to 900 nm: more or less very similar responses are observed for all devices. It can be seen in the figure that for all devices, the quantum efficiency rises from photon wavelengths greater than 300 nm, while the highest QE is achieved in the 480–485 nm range of photon wavelengths for all devices; then, the QE of devices gradually decreased, and the relative magnified quantum efficiency responses for all devices are shown in the inset of Figure 12. This clearly reveals that the device, which uses BT-BIC as its electron transport layer, exhibits relatively improved quantum efficiency, and it also confirms the earlier simulation results that were already discussed.

In addition, Figure 12 shows that the quantum efficiency of the BT-BIC device reaches its optimum value, even though the short-circuit current does not. As shown in Figure 11, the device BT-L4F displays the maximum short-circuit current. While there is a correlation between short-circuit current and quantum efficiency, it is essential to recognize that recombination losses can lead to such discrepancies. Quantum efficiency refers to the measurement of the effectiveness of photon absorption and subsequent transformation into charge carriers. In contrast, the short-circuit current density includes other variables, such as recombination losses, resistive losses, and other internal variables. Consequently, the presence of these losses may cause a slight decrease in the short-circuit current relative to the theoretical quantum efficiency. Given the comparative nature of this study, it can be argued that while the BT-LIC device has a slightly lower short-circuit current than the BT-L4F device, it has superior electrical and optical properties. Figure 11 depicts the enhanced photovoltaic response as evidence.

Based on the data presented in Table 3, it is evident that a majority of the efficient NFA electron transport layers have been reported in association with the leading MAPbI_3_-based perovskite solar cells, which are toxic in nature due to the presence of Pb. The power conversion efficiency of other lead-free perovskites (having very close energy bandgaps compared to Cs_2_AgBi_0.75_Sb_0.25_Br_6_) with an NFA electron transport layer is not up to the mark, as evident in Table 3. Comparing innovative electron transport layer materials, such as BT-LIC, BT-BIC, BT-L4F, and BT-BO-L4F, for perovskite Cs_2_AgBi_0.75_Sb_0.25_Br_6_-based solar cells with previously reported NFA-based lead-free perovskite solar cells, as shown in Table 3, poses significant improvements. Such improvements may be due to their distinctive electronic characteristics and customized energy levels, which enable effective charge extraction and transportation throughout the device.

The electron transport layers (ETLs), namely BT-LIC, BT-BIC, BT-L4F, and BT-BO-L4F, are likely comprised of organic or mixed materials. These materials seek to facilitate charge transport within the electron transport layer for Cs_2_AgBi_0.75_Sb_0.25_Br_6_-based lead-free perovskite materials, which are still not so efficient. This is because the mobility of the abovementioned ETL, which falls within the range of 10^−^^4^ cm^2^/V.sec, is lower when compared to the mobility of C60-based thin films, which is measured at 0.5 ± 0.2 cm^2^/V.sec. The limited mobility exhibited by these non-fullerene acceptors (NFAs) has the potential to lower the efficiency of electron collection, increase recombination rates, and contribute to increased series resistance. Consequently, all these factors collectively contribute to the deterioration of the photovoltaic responses of the proposed devices.

The use of innovative electron transport layers, including BT-LIC, BT-BIC, BT-L4F, and BT-BO-L4F, in perovskite solar cells has resulted in a notable improvement in power conversion efficiency when compared to other lead-free solar cell technologies based on non-fullerene acceptors (NFAs). The use of the novel BT-BIC in ETL materials and design facilitates increased photon absorption and charge carrier mobility, hence leading to improved power conversion efficiency. Based on our limited information, no experimental or even modeling findings have been published for the use of the innovative above-listed electron transport layers with Cs_2_AgBi_0.75_Sb_0.25_Br_6_-based perovskite in the context of solar cell applications. In general, the research exhibits significant promise in terms of developing the domain of solar energy harvesting.

## 7. Conclusions

In this comparative study, four non-fullerene acceptors (NFAs), (i) BT-LIC, (ii) BT-BIC, (iii) BT-L4F, and (iv) BT-BO-L4F, were used as electron transport layers for novel proposed solar devices, i.e., FTO/PEDOT: PSS/Cs_2_AgBi_0.75_Sb_0.25_Br_6_/ETL/Au. All these devices were comprehensively investigated through simulation to determine the most efficient electron transport layer for the proposed devices. For this purpose, comprehensive simulations were carried out to optimize each layer with respect to film thickness and doping density, and then the photovoltaic responses of all the optimized devices were simulated as a function of the electron transport layer, and open-circuit voltage, short-circuit current, fill factor, and power conversion efficiency were determined. It is observed that all devices show reasonable photovoltaic responses, and the device containing BT-BIC as an electron transport layer shows the highest power conversion efficiency of ~13.2% (open-circuit voltage = ~1.36 V, short-circuit current = ~12.1 mA/cm^2^, and fill factor = ~80%). The BT-LIC device shows the lowest power conversion efficiency of approximately ~6.8% (open-circuit voltage = ~1.23 V, short-circuit current = ~11.2 mA/cm^2^, and fill factor = ~50%). It is also observed that the improvements in open-circuit voltage and fill factor are relatively higher for device B. This clearly demonstrates that the photovoltaic device B fabricated with BT-BIC as an electron transport layer may manage high build-in potential and excellent interface quality, which in turn improve the overall charge collection and charge transport and suppress recombination losses compared to the other electron transport layers BT-LIC, BT-L4F, and BT-BO-L4F.

## Figures and Tables

**Table 2:** Optimized photovoltaic responses of (i) open-circuit voltage, (ii) short-circuit current, (iii) fill factor, and (iv) power conversion efficiency of the proposed (a) device BT-LIC, (b) device BT-BIC, (c) device BT-L4F, and (d) device BT-BO-L4F.

Device	Open-Circuit Voltage (Volts)	Short-Circuit Current (mA.cm^−2^)	Fill Factor (%)	Power Conversion Efficiency (%)
Device BT-LIC	1.23	11.2	50	6.8
Device BT-BIC	1.36	12.1	80	13.2
Device BT-L4F	1.26	12.01	71	10.7
Device BT-BO-L4F	1.24	12.5	78	12.09

**Table 3:** Comparing innovative electron transport layer materials, such as BT-LIC, BT-BIC, BT-L4F, and BT-BO-L4F, for perovskite Cs2AgBi0.75Sb0.25Br6-based solar cells with previously reported solar cells.

Perovskite Layer	Hole Transport Layer	Electron Transport Layer (NFA)	Short-Circuit Current	Open-Circuit Voltage	Fill Factor	Power Conversion Efficiency	Ref.
MAPbI_3_	PEDOT:PSS	PDI	6.65	0.64	15.4	0.66	[90]
MAPbI_3_	PEDOT:PSS	PDI	14.64	0.75	29.5	3.23	[90]
MAPbI_3_	NiOx	HATNA	10.17	0.85	57.6	4.69	[91]
MAPbI_3_	PEDOT:PSS	BPTI	15.28	0.88	56.04	7.54	[92]
MAPbI_3_	PCDTBT	PDI2	21.29	1.06	69.57	15.75	[93]
MAPbI_3_	PEDOT:PSS	NDI-PM	21.2	1.1	79.1	18.4	[94]
(MA_0.8_FA_0.2_) Pb(I_0.93_Cl_0.07_)_3_	PEDOT:PSS	NDIF3	22.11	0.91	56	11.17	[94]
MAPbI_3−x_Cl_x_	PEDOT:PSS	PDPT	22.9	0.76	44	7.6	[23]
MAPbI_3_	P3CT	IT-4M	21.84	1.086	79.75	18.92	[95]
MAPbI_3_	NiOx	TPA-3CN	22.5	1.05	81.1	19.2	[96]
Cs_3_Bi_2_I_9_	P3HT	TiO2	0.34	0.31	38	0.4	[97]
Cs_2_AgBiBr_6_	PEDOT:PSS	ZnO	11.2	1.05	43.97	5.16	[98]
Cs_2_AgBiBr_6_	Cu_2_O	ZnO	11.2	0.972	47.43	5.15	[98]
Cs_2_AgBiBr_6_	P_3_HT	ZnO	11.1027	0.92	44.02	4.48	[99]
BA_2_MA_3_Sn_4_I_13_	PTAA	TiO_2_	24.1	0.229	45.7	2.53	[100]
MASnI_3-x_Br_x_	PTAA	TiO_2_	0.452	5.02	48.3	1.10	[101]
Cs_2_AgBiBr_6_	PTAA	TiO_2_	1.24	1.06	78	1.02	[102]
Cs_2_AgBi_0.875_Sb_0.125_Br_6_	PTAA	TiO_2_	0.94	0.59	51	0.28	[102]
Cs_2_AgBi_0.8_Sb_0.2_Br_6_	PTAA	TiO_2_	0.48	0.78	44	0.16	[102]
Cs_2_AgBi_0.75_Sb_0.25_Br_6_	PTAA	TiO_2_	0.55	0.25	50	0.08	[102]
Cs_2_AgBi_0.75_Sb_0.25_Br_6_	PEDOT:PSS	BT-LIC	11.2	1.23	50	6.8	This Study
Cs_2_AgBi_0.75_Sb_0.25_Br_6_	PEDOT:PSS	BT-BIC	12.1	1.36	80	13.2	
Cs_2_AgBi_0.75_Sb_0.25_Br_6_	PEDOT:PSS	BT-L4F	12.01	1.26	71	10.7	
Cs_2_AgBi_0.75_Sb_0.25_Br_6_	PEDOT:PSS	BT-BO-L4F	12.5	1.24	78	12.09	

## Data Availability

The data presented in this study are available on request from the corresponding author.

## References

[1] Liu S., Biju V.P., Qi Y., Chen W., Liu Z. (2023). Recent Progress in the Development of High-Efficiency Inverted Perovskite Solar Cells. NPG Asia Mater..

[2] Shao M., Bie T., Yang L., Gao Y., Jin X., He F., Zheng N., Yu Y., Zhang X. (2022). Over 21% Efficiency Stable 2D Perovskite Solar Cells. Adv. Mater..

[3] Rong Y., Hu Y., Mei A., Tan H., Saidaminov M.I., Seok S., McGehee M.D., Sargent E.H., Han H. (2018). Challenges for Commercializing Perovskite Solar Cells. Science.

[4] Liu J., Aydin E., Yin J., De Bastiani M., Isikgor F.H., Rehman A.U., Yengel E., Ugur E., Harrison G.T., Wang M. (2021). 28.2%-Efficient, Outdoor-Stable Perovskite/Silicon Tandem Solar Cell. Joule.

[5] Babayigit A., Ethirajan A., Muller M., Conings B. (2016). Toxicity of Organometal Halide Perovskite Solar Cells. Nat. Mater..

[6] Ding G., Zheng Y., Xiao X., Cheng H., Zhang G., Shi Y., Shao Y. (2022). Sustainable Development of Perovskite Solar Cells: Keeping a Balance between Toxicity and Efficiency. J. Mater. Chem. A.

[7] Moiz S.A., Alahmadi A.N.M., Alshaikh M.S. (2023). Lead-Free FACsSnI3 Based Perovskite Solar Cell: Designing Hole and Electron Transport Layer. Nanomaterials.

[8] Xu F., Zhang M., Li Z., Yang X., Zhu R. (2023). Challenges and Perspectives toward Future Wide-Bandgap Mixed-Halide Perovskite Photovoltaics. Adv. Energy Mater..

[9] Bello O.O., Emetere M.E. (2022). Progress and Limitation of Lead-Free Inorganic Perovskites for Solar Cell Application. Solar Energy.

[10] Yu W., Zou Y., Wang H., Qu B., Chen Z., Xiao L. (2023). Expanding the Absorption of Double Perovskite Cs2AgBiBr6 to NIR Region. J. Phys. Chem. Lett..

[11] Zhai M., Chen C., Cheng M. (2023). Advancing Lead-Free Cs2AgBiBr6 Perovskite Solar Cells: Challenges and Strategies. Solar Energy.

[12] Igbari F., Xu F.F., Shao J.Y., Ud-Din F., Siffalovic P., Zhong Y.W. (2023). Stacking Interactions and Photovoltaic Performance of Cs2AgBiBr6 Perovskite. Solar RRL.

[13] Momblona C., Gil-Escrig L., Bandiello E., Hutter E.M., Sessolo M., Lederer K., Blochwitz-Nimoth J., Bolink H.J. (2016). Efficient Vacuum Deposited P-i-n and n-i-p Perovskite Solar Cells Employing Doped Charge Transport Layers. Energy Environ. Sci..

[14] Correa-Baena J.P., Saliba M., Buonassisi T., Grätzel M., Abate A., Tress W., Hagfeldt A. (2017). Promises and Challenges of Perovskite Solar Cells. Science.

[15] Foo S., Thambidurai M., Senthil Kumar P., Yuvakkumar R., Huang Y., Dang C. (2022). Recent Review on Electron Transport Layers in Perovskite Solar Cells. Int. J. Energy Res..

[16] Mohamad Noh M.F., Teh C.H., Daik R., Lim E.L., Yap C.C., Ibrahim M.A., Ahmad Ludin N., bin Mohd Yusoff A.R., Jang J., Mat Teridi M.A. (2018). The Architecture of the Electron Transport Layer for a Perovskite Solar Cell. J. Mater. Chem. C.

[17] Mihailetchi V.D., Van Duren J.K.J., Blom P.W.M., Hummelen J.C., Janssen R.A.J., Kroon J.M., Rispens M.T., Verhees W.J.H., Wienk M.M. (2003). Electron Transport in a Methanofullerene. Adv. Funct. Mater..

[18] Yang D., Zhang X., Wang K., Wu C., Yang R., Hou Y., Jiang Y., Liu S., Priya S. (2019). Stable Efficiency Exceeding 20.6% for Inverted Perovskite Solar Cells through Polymer-Optimized PCBM Electron-Transport Layers. Nano Lett..

[19] Karuppuswamy P., Hanmandlu C., Moorthy Boopathi K., Perumal P., Liu C., Chen Y.-F., Chang Y.-C., Wang P.-C., Lai C.-S., Chu C.-W. (2017). Solution-Processable Electron Transport Layer for Efficient Hybrid Perovskite Solar Cells beyond Fullerenes. Sol. Energy Mater. Sol. Cells.

[20] Saki Z., Aitola K., Sveinbjörnsson K., Yang W., Svanström S., Cappel U.B., Rensmo H., Johansson E.M.J., Taghavinia N., Boschloo G. (2018). The Synergistic Effect of Dimethyl Sulfoxide Vapor Treatment and C60 Electron Transporting Layer towards Enhancing Current Collection in Mixed-Ion Inverted Perovskite Solar Cells. J. Power Sour..

[21] Etxebarria I., Ajuria J., Pacios R. (2015). Polymer:Fullerene Solar Cells: Materials, Processing Issues, and Cell Layouts to Reach Power Conversion Efficiency over 10%, a Review. J. Photon. Energy.

[22] Wang D., Ye T., Zhang Y. (2020). Recent Advances of Non-Fullerene Organic Electron Transport Materials in Perovskite Solar Cells. J. Mater. Chem. A Mater..

[23] Heo J.H., Lee S.-C., Jung S.-K., Kwon O.-P., Im S.H. (2017). Efficient and Thermally Stable Inverted Perovskite Solar Cells by Introduction of Non-Fullerene Electron Transporting Materials. J. Mater. Chem. A Mater..

[24] Padula D., Landi A., Prampolini G. (2023). Assessing Alkyl Side Chain Effects on Electron Transport Properties of Y6-Derived Non-Fullerene Acceptors. Energy Adv..

[25] Cao X., Li P., Zhu X., Li H., Xu R., Li J., Ma L., Dong H., Wu Z. (2023). Nonfullerene Agent Enables Efficient and Stable Tin-Based Perovskite Solar Cells. Solar RRL.

[26] Lin Y., Wang J., Zhang Z.G., Bai H., Li Y., Zhu D., Zhan X. (2015). An Electron Acceptor Challenging Fullerenes for Efficient Polymer Solar Cells. Adv. Mater..

[27] Zhou D., Wang J., Xu Z., Xu H., Quan J., Deng J., Li Y., Tong Y., Hu B., Chen L. (2022). Recent Advances of Nonfullerene Acceptors in Organic Solar Cells. Nano Energy.

[28] Wei Q., Liu W., Leclerc M., Yuan J., Chen H., Zou Y. (2020). A-DA′D-A Non-Fullerene Acceptors for High-Performance Organic Solar Cells. Sci. China Chem..

[29] Yuan J., Zhang Y., Zhou L., Zhang G., Yip H.-L., Lau T.-K., Lu X., Zhu C., Peng H., Johnson P.A. (2019). Single-Junction Organic Solar Cell with over 15% Efficiency Using Fused-Ring Acceptor with Electron-Deficient Core. Joule.

[30] Cui Y., Yao H., Zhang J., Xian K., Zhang T., Hong L., Wang Y., Xu Y., Ma K., An C. (2020). Single-Junction Organic Photovoltaic Cells with Approaching 18% Efficiency. Adv. Mater..

[31] Zhu C., Yuan J., Cai F., Meng L., Zhang H., Chen H., Li J., Qiu B., Peng H., Chen S. (2020). Tuning the Electron-Deficient Core of a Non-Fullerene Acceptor to Achieve over 17% Efficiency in a Single-Junction Organic Solar Cell. Energy Environ. Sci..

[32] Cai Y., Li Y., Wang R., Wu H., Chen Z., Zhang J., Ma Z., Hao X., Zhao Y., Zhang C. (2021). A Well-Mixed Phase Formed by Two Compatible Non-Fullerene Acceptors Enables Ternary Organic Solar Cells with Efficiency over 18.6%. Adv. Mater..

[33] Liu Q., Jiang Y., Jin K., Qin J., Xu J., Li W., Xiong J., Liu J., Xiao Z., Sun K. (2020). 18% Efficiency Organic Solar Cells. Sci. Bullet..

[34] Jin K., Xiao Z., Ding L. (2021). 18.69% PCE from Organic Solar Cells. J. Semicond..

[35] Li S., Ye L., Zhao W., Zhang S., Mukherjee S., Ade H., Hou J. (2016). Energy-Level Modulation of Small-Molecule Electron Acceptors to Achieve over 12% Efficiency in Polymer Solar Cells. Adv. Mater..

[36] Lin Y., He Q., Zhao F., Huo L., Mai J., Lu X., Su C.J., Li T., Wang J., Zhu J. (2016). A Facile Planar Fused-Ring Electron Acceptor for As-Cast Polymer Solar Cells with 8.71% Efficiency. J. Am. Chem. Soc..

[37] Pan H., Zhao X., Gong X., Li H., Ladi N.H., Zhang X.L., Huang W., Ahmad S., Ding L., Shen Y. (2020). Advances in Design Engineering and Merits of Electron Transporting Layers in Perovskite Solar Cells. Mater. Horiz..

[38] Mahmood K., Sarwar S., Mehran M.T. (2017). Current Status of Electron Transport Layers in Perovskite Solar Cells: Materials and Properties. RSC Adv..

[39] Saif O.M., Elogail Y., Abdolkader T.M., Shaker A., Zekry A., Abouelatta M., Salem M.S., Fedawy M. (2023). Comprehensive Review on Thin Film Homojunction Solar Cells: Technologies, Progress and Challenges. Energies.

[40] Bagade S.S., Barik S.B., Malik M.M., Patel P.K. (2023). Impact of Band Alignment at Interfaces in Perovskite-Based Solar Cell Devices. Mater. Today Proc..

[41] Ma L., Zhang S., Hou J. (2022). Crystal Structures in State-of-the-Art Non-Fullerene Electron Acceptors. J. Mater. Chem. A.

[42] Li G., Zhang X., Jones L.O., Alzola J.M., Mukherjee S., Feng L.W., Zhu W., Stern C.L., Huang W., Yu J. (2021). Systematic Merging of Nonfullerene Acceptor π-Extension and Tetrafluorination Strategies Affords Polymer Solar Cells with >16% Efficiency. J. Am. Chem. Soc..

[43] Xin J., Li W., Zhang Y., Liang Q., Song C., Zhao Y., He Z., Liu J., Ma W. (2023). A Review of Nonfullerene Solar Cells: Insight into the Correlation among Molecular Structure, Morphology, and Device Performance. Battery Energy.

[44] Liang C., Xing G. (2021). Doping Electron Transporting Layer: An Effective Method to Enhance JSC of All-Inorganic Perovskite Solar Cells. Energy Environ. Mater..

[45] Lee S., Paine D.C., Gleason K.K. (2014). Heavily Doped Poly(3,4-Ethylenedioxythiophene) Thin Films with High Carrier Mobility Deposited Using Oxidative CVD: Conductivity Stability and Carrier Transport. Adv. Funct. Mater..

[46] Xu Z., Li N., Niu X., Liu H., Liu G., Chen Q., Zhou H. (2022). Balancing Energy-Level Difference for Efficient n-i-p Perovskite Solar Cells with Cu Electrode. Energy Mater. Adv..

[47] Wu X., Li B., Zhu Z., Chueh C.C., Jen A.K.Y. (2021). Designs from Single Junctions, Heterojunctions to Multijunctions for High-Performance Perovskite Solar Cells. Chem. Soc. Rev..

[48] Zhang S., Liu Z., Zhang W., Jiang Z., Chen W., Chen R., Huang Y., Yang Z., Zhang Y., Han L. (2020). Barrier Designs in Perovskite Solar Cells for Long-Term Stability. Adv. Energy Mater..

[49] Fan R., Huang Y., Wang L., Li L., Zheng G., Zhou H. (2016). The Progress of Interface Design in Perovskite-Based Solar Cells. Adv. Energy Mater..

[50] Hossain M.K., Samajdar D.P., Das R.C., Arnab A.A., Rahman M.F., Rubel M.H.K., Islam M.R., Bencherif H., Pandey R., Madan J. (2023). Design and Simulation of Cs2BiAgI6 Double Perovskite Solar Cells with Different Electron Transport Layers for Efficiency Enhancement. Energy Fuels.

[51] Mali S.S., Hong C.K. (2016). P-i-n/p-Type Planar Hybrid Structure of Highly Efficient Perovskite Solar Cells towards Improved Air Stability: Synthetic Strategies and the Role of p-Type Hole Transport Layer (HTL) and n-Type Electron Transport Layer (ETL) Metal Oxides. Nanoscale.

[52] Wu S., Liu M., Jen A.K.Y. (2023). Prospects and Challenges for Perovskite-Organic Tandem Solar Cells. Joule.

[53] Sun K., Zhang S., Li P., Xia Y., Zhang X., Du D., Isikgor F.H., Ouyang J. (2015). Review on Application of PEDOTs and PEDOT:PSS in Energy Conversion and Storage Devices. J. Mater. Sci. Mater. Electron..

[54] Liu F., Zhu J., Wei J., Li Y., Lv M., Yang S., Zhang B., Yao J., Dai S. (2014). Numerical Simulation: Toward the Design of High-Efficiency Planar Perovskite Solar Cells. Appl. Phys. Lett..

[55] Neukom M.T., Schiller A., Züfle S., Knapp E., Ávila J., Pérez-del-Rey D., Dreessen C., Zanoni K.P.S., Sessolo M., Bolink H.J. (2019). Consistent Device Simulation Model Describing Perovskite Solar Cells in Steady-State, Transient, and Frequency Domain. ACS Appl. Mater. Interfaces.

[56] Gummel H.K. (1964). A Self-Consistent Ierative Scheme for One-Dimensional Steady State Transistor Calculations. IEEE Trans. Electron. Dev..

[57] Gwyn C.W., Scharfetter D.L., Wirth J.L. (1967). The Analysis of Radiation Effects in Semiconductor Junction Devices. IEEE Trans. Nucl. Sci..

[58] Burgelman M., Decock K., Niemegeers A., Verschraegen J., Degrave S. (2016). SCAPS Manual.

[59] Burgelman M., Nollet P., Degrave S. (2000). Modelling Polycrystalline Semiconductor Solar Cells. Thin Solid Films.

[60] Liu Y., Sun Y., Rockett A. (2012). A New Simulation Software of Solar Cells—WxAMPS. Sol. Energy Mater. Sol. Cells.

[61] Moiz S.A. (2022). Optimization of Hole and Electron Transport Layer for Highly Efficient Lead-Free Cs2TiBr6-Based Perovskite Solar Cell. Photonics.

[62] Moiz S.A., Alahmadi A.N.M. (2021). Design of Dopant and Lead-Free Novel Perovskite Solar Cell for 16.85% Efficiency. Polymers.

[63] Burgelman M., Decock K., Khelifi S., Abass A. (2013). Advanced Electrical Simulation of Thin Film Solar Cells. Thin Solid Films.

[64] Burgelman M., Verschraegen J., Degrave S., Nollet P. (2004). Modeling Thin-Film PV Devices. Prog. Photovolt. Res. Appl..

[65] Verschraegen J., Burgelman M. (2007). Numerical Modeling of Intra-Band Tunneling for Heterojunction Solar Cells in Scaps. Thin Solid Films.

[66] He Y., Xu L., Yang C., Guo X., Li S. (2021). Design and Numerical Investigation of a Lead-Free Inorganic Layered Double Perovskite Cs4cusb2cl12 Nanocrystal Solar Cell by Scaps-1d. Nanomaterials.

[67] Chowdhury M.S., Shahahmadi S.A., Chelvanathan P., Tiong S.K., Amin N., Techato K., Nuthammachot N., Chowdhury T., Suklueng M. (2020). Effect of Deep-Level Defect Density of the Absorber Layer and n/i Interface in Perovskite Solar Cells by SCAPS-1D. Results Phys..

[68] Moiz S.A., Albadwani S.A., Alshaikh M.S. (2022). Towards Highly Efficient Cesium Titanium Halide Based Lead-Free Double Perovskites Solar Cell by Optimizing the Interface Layers. Nanomaterials.

[69] Moiz S.A., Alzahrani M.S., Alahmadi A.N.M. (2022). Electron Transport Layer Optimization for Efficient PTB7:PC70BM Bulk-Heterojunction Solar Cells. Polymers.

[70] Moiz S.A., Alahmadi A.N.M., Aljohani A.J. (2021). Design of a Novel Lead-Free Perovskite Solar Cell for 17.83% Efficiency. IEEE Access.

[71] McClure E.T., Ball M.R., Windl W., Woodward P.M. (2016). Cs2AgBiX6 (X = Br, Cl): New Visible Light Absorbing, Lead-Free Halide Perovskite Semiconductors. Chem. Mater..

[72] Amri K., Belghouthi R., Aillerie M., Gharbi R. (2021). Device Optimization of a Lead-Free Perovskite/Silicon Tandem Solar Cell with 24.4% Power Conversion Efficiency. Energies.

[73] Zhang G., Lin F.R., Qi F., Heumüller T., Distler A., Egelhaaf H.J., Li N., Chow P.C.Y., Brabec C.J., Jen A.K.Y. (2022). Renewed Prospects for Organic Photovoltaics. Chem. Rev..

[74] Liu H., Dai T., Zhou J., Wang H., Guo Q., Guo Q., Zhou E. (2023). The Development of A-DA’D-A Type Nonfullerene Acceptors Containing Non-Halogenated End Groups. Nano Res..

[75] Perdigón-Toro L., Zhang H., Markina A., Yuan J., Hosseini S.M., Wolff C.M., Zuo G., Stolterfoht M., Zou Y., Gao F. (2020). Barrierless Free Charge Generation in the High-Performance PM6:Y6 Bulk Heterojunction Non-Fullerene Solar Cell. Adv. Mater..

[76] Shi T., Zhang H.S., Meng W., Teng Q., Liu M., Yang X., Yan Y., Yip H.L., Zhao Y.J. (2017). Effects of Organic Cations on the Defect Physics of Tin Halide Perovskites. J. Mater. Chem. A Mater..

[77] Cao W., Hu Z., Lin Z., Guo X., Su J., Chang J., Hao Y. (2022). Defects and Doping Engineering towards High Performance Lead-Free or Lead-Less Perovskite Solar Cells. J. Energy Chem..

[78] Friedel B., Keivanidis P.E., Brenner T.J.K., Abrusci A., McNeill C.R., Friend R.H., Greenham N.C. (2009). Effects of Layer Thickness and Annealing of PEDOT:PSS Layers in Organic Photodetectors. Macromolecules.

[79] Moiz S.A., Khan I.A., Younis W.A., Masud M.I., Ismail Y., Khawaja Y.M. (2020). Solvent Induced Charge Transport Mechanism for Conducting Polymer at Higher Temperature. Mater. Res. Express.

[80] Karimov K.S., Ahmed M.M., Moiz S.A., Babadzhanov P., Marupov R., Turaeva M.A. (2003). Electrical Properties of Organic Semiconductor Orange Nitrogen Dye Thin Films Deposited from Solution at High Gravity. Eurasian Chem.-Technol. J..

[81] Sherkar T.S., Momblona C., Gil-Escrig L., Ávila J., Sessolo M., Bolink H.J., Koster L.J.A. (2017). Recombination in Perovskite Solar Cells: Significance of Grain Boundaries, Interface Traps, and Defect Ions. ACS Energy Lett..

[82] Moiz S.A., Alahmadi A.N.M., Aljohani A.J. (2020). Design of Silicon Nanowire Array for PEDOT:PSS-Silicon Nanowire-Based Hybrid Solar Cell. Energies.

[83] Della Gaspera E., Peng Y., Hou Q., Spiccia L., Bach U., Jasieniak J.J., Cheng Y.B. (2015). Ultra-Thin High Efficiency Semitransparent Perovskite Solar Cells. Nano Energy.

[84] Brendel R., Queisser H.J. (1993). On the Thickness Dependence of Open Circuit Voltages of P-n Junction Solar Cells. Solar Energy Mater. Solar Cells.

[85] Zhang C., Zhu X. (2020). N-Type Quinoidal Oligothiophene-Based Semiconductors for Thin-Film Transistors and Thermoelectrics. Adv. Funct. Mater..

[86] Naab B.D., Gu X., Kurosawa T., To J.W.F., Salleo A., Bao Z. (2016). Role of Polymer Structure on the Conductivity of N-Doped Polymers. Adv. Electron. Mater..

[87] Moiz S.A., Alahmadi A.N.M., Karimov K.S. (2020). Improved Organic Solar Cell by Incorporating Silver Nanoparticles Embedded Polyaniline as Buffer Layer. Solid State Electron..

[88] Guo Z., Jena A.K., Kim G.M., Miyasaka T. (2022). The High Open-Circuit Voltage of Perovskite Solar Cells: A Review. Energy Environ. Sci..

[89] Shao Y., Yuan Y., Huang J. (2016). Correlation of Energy Disorder and Open-Circuit Voltage in Hybrid Perovskite Solar Cells. Nat. Energy.

[90] Shaheen S.E., Brabec C.J., Sariciftci N.S., Padinger F., Fromherz T., Hummelen J.C. (2001). 2.5% Efficient Organic Plastic Solar Cells. Appl. Phys. Lett..

[91] Wu J., Huang W.-K., Chang Y.-C., Tsai B.-C., Hsiao Y.-C., Chang C.-Y., Chen C.-T., Chen C.-T. (2017). Simple Mono-Halogenated Perylene Diimides as Non-Fullerene Electron Transporting Materials in Inverted Perovskite Solar Cells with ZnO Nanoparticle Cathode Buffer Layers. J. Mater. Chem. A Mater..

[92] Angmo D., Peng X., Cheng J., Gao M., Rolston N., Sears K., Zuo C., Subbiah J., Kim S.-S., Weerasinghe H. (2018). Beyond Fullerenes: Indacenodithiophene-Based Organic Charge-Transport Layer toward Upscaling of Low-Cost Perovskite Solar Cells. ACS Appl. Mater. Interfaces.

[93] Karuppuswamy P., Chen H.-C., Wang P.-C., Hsu C.-P., Wong K.-T., Chu C.-W. (2018). The 3 D Structure of Twisted Benzo[Ghi]Perylene-Triimide Dimer as a Non-Fullerene Acceptor for Inverted Perovskite Solar Cells. ChemSusChem.

[94] Wang P., Cai F., Yang L., Yan Y., Cai J., Wang H., Gurney R.S., Liu D., Wang T. (2018). Eliminating Light-Soaking Instability in Planar Heterojunction Perovskite Solar Cells by Interfacial Modifications. ACS Appl. Mater. Interfaces.

[95] Liu X., Li X., Zou Y., Liu H., Wang L., Fang J., Yang C. (2019). Energy Level-Modulated Non-Fullerene Small Molecule Acceptors for Improved VOC and Efficiency of Inverted Perovskite Solar Cells. J. Mater. Chem. A Mater..

[96] Chen C., Li H., Ding X., Cheng M., Li H., Xu L., Qiao F., Li H., Sun L. (2018). Molecular Engineering of Triphenylamine-Based Non-Fullerene Electron-Transport Materials for Efficient Rigid and Flexible Perovskite Solar Cells. ACS Appl. Mater. Interfaces.

[97] Ran C., Wu Z., Xi J., Yuan F., Dong H., Lei T., He X., Hou X. (2017). Construction of Compact Methylammonium Bismuth Iodide Film Promoting Lead-Free Inverted Planar Heterojunction Organohalide Solar Cells with Open-Circuit Voltage over 0.8 V. J. Phys. Chem. Lett..

[98] Alam I., Mollick R., Ashraf M.A. (2021). Numerical Simulation of Cs2AgBiBr6-Based Perovskite Solar Cell with ZnO Nanorod and P3HT as the Charge Transport Layers. Phys. B Condens. Matter..

[99] Rai S., Pandey B.K., Garg A., Dwivedi D.K. (2021). Hole transporting layer optimization for an efficient lead-free double perovskite solar cell by numerical simulation. Opt. Mater..

[100] Cao D.H., Stoumpos C.C., Yokoyama T., Logsdon J.L., Song T.-B., Farha O.K., Wasielewski M.R., Hupp J.T., Kanatzidis M.G. (2017). Thin Films and Solar Cells Based on Semiconducting Two-Dimensional Ruddlesden–Popper (CH3(CH2)3NH3)2(CH3NH3)N−1SnnI3n+1 Perovskites. ACS Energy Lett..

[101] Wang N., Zhou Y., Ju M.G., Garces H.F., Ding T., Pang S., Zeng X.C., Padture N.P., Sun X.W. (2016). Heterojunction-Depleted Lead-Free Perovskite Solar Cells with Coarse-Grained B-γ-CsSnI3 Thin Films. Adv. Energy Mater..

[102] Pantaler M., Olthof S., Meerholz K., Lupascu D.C. (2019). Bismuth-Antimony Mixed Double Perovskites Cs2AgBi1-XSbxBr6in Solar Cells. MRS Adv..

